# Supplementary material for: An anchoring complex recruits katanin for microtubule severing at the plant cortical nucleation sites
Source: Nat Commun. 2021 Jun 17;12:3687. doi: 10.1038/s41467-021-24067-y (PMC8211667; doi:10.1038/s41467-021-24067-y)
Supplement: Supplementary file 1 — Supplementary Information [file 41467_2021_24067_MOESM1_ESM.pdf]

a

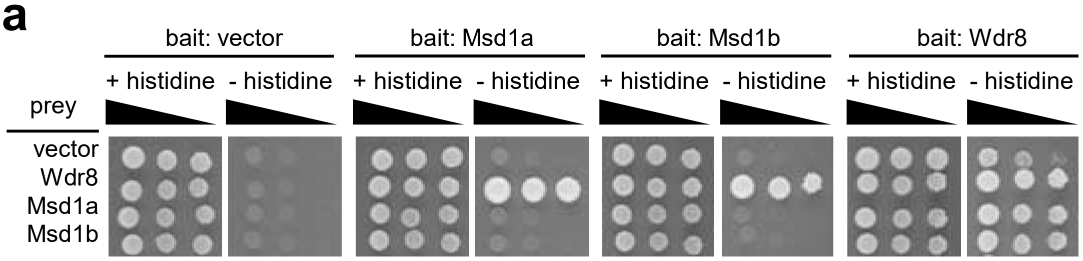

b

Pull-down results from Msd1b-GFP-expressing plants

position	Matched protein	score	Matched peptides	Coverage (%)
1	Msd1b	1,632	95	73
2	Wdr8	860	42	65
3	Msd1a	499	26	37
3	Msd1b	1,213	64	72

Pull-down results from Wdr8-GFP-expressing plants

position	Matched protein	score	Matched peptides	Coverage (%)
4	Wdr8	1,207	73	75
5	Wdr8	63	5	12
6	Msd1a	548	39	65
6	Msd1b	516	31	43

Supplementary Figure 1. Msd1 and Wdr8 form complexes. **a**, Interactions of Msd1a, Msd1b and Wdr8 with themselves in yeast two-hybrid assays. Yeast cells were diluted (from left to right: no dilution, 1/10 dilution, and 1/100 dilution) and grown on a control medium (+histidine) or a selection (-histidine) medium. **b**, LC-MS/MS analysis of pulled-down proteins. Soluble proteins were immuno-precipitated by a GFP antibody from Arabidopsis seedlings expressing Msd1b-GFP or Wdr8-GFP. Proteins in the regions labeled with the numbers in SDS-PAGE gels (Figure 1a) were recovered and analyzed separately.

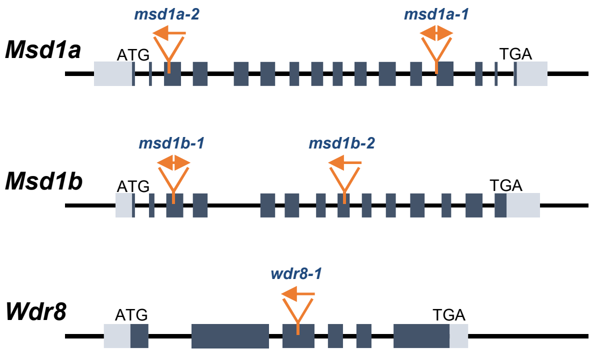

Supplementary Figure 2. T-DNA insertion mutant alleles at the loci of *Msd1*a, *Msd1b*, and *Wdr8*. The black and grey boxes respectively indicate coding and non-coding regions, whereas the lines show intergenic regions and introns. T-DNAs are indicated by arrows with the left borders face the pointed ends of the arrowheads. Double headed arrows indicate that two copies of inverted T-DNAs are integrated.

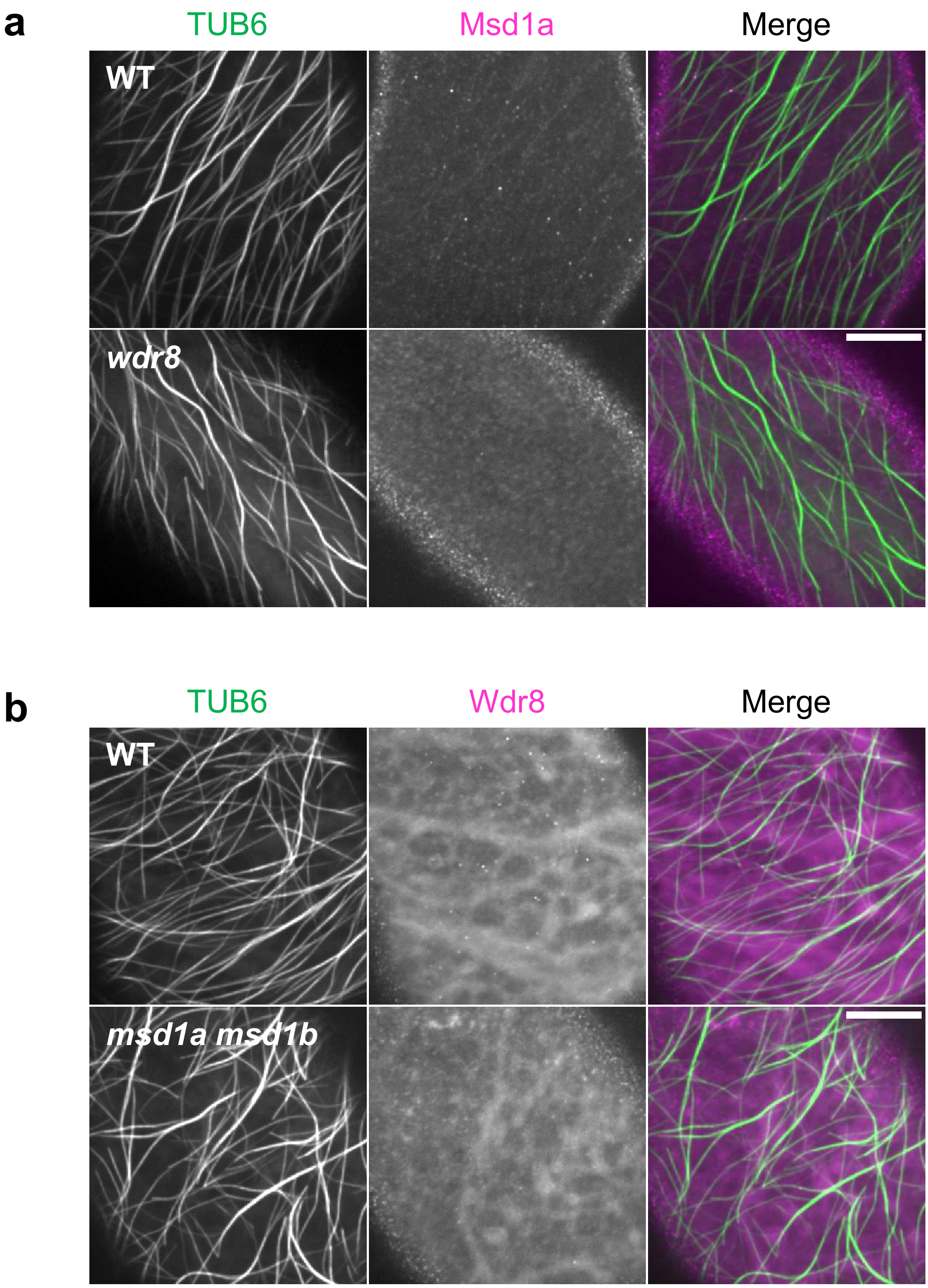

Supplementary Figure 3. Recruitment of Msd1a-GFP particles (a) and Wdr8-GFP particles (b) to the cortical region of cotyledon epidermal cells. Averaged projection images of 302 s in wild type (above), *wdr8* (**a**, below), and *msd1a msd1b* (**b**, below) cells are shown. Cortical microtubules are labeled with the mCherry-TUB6 marker (green). Bars, 10 μm.

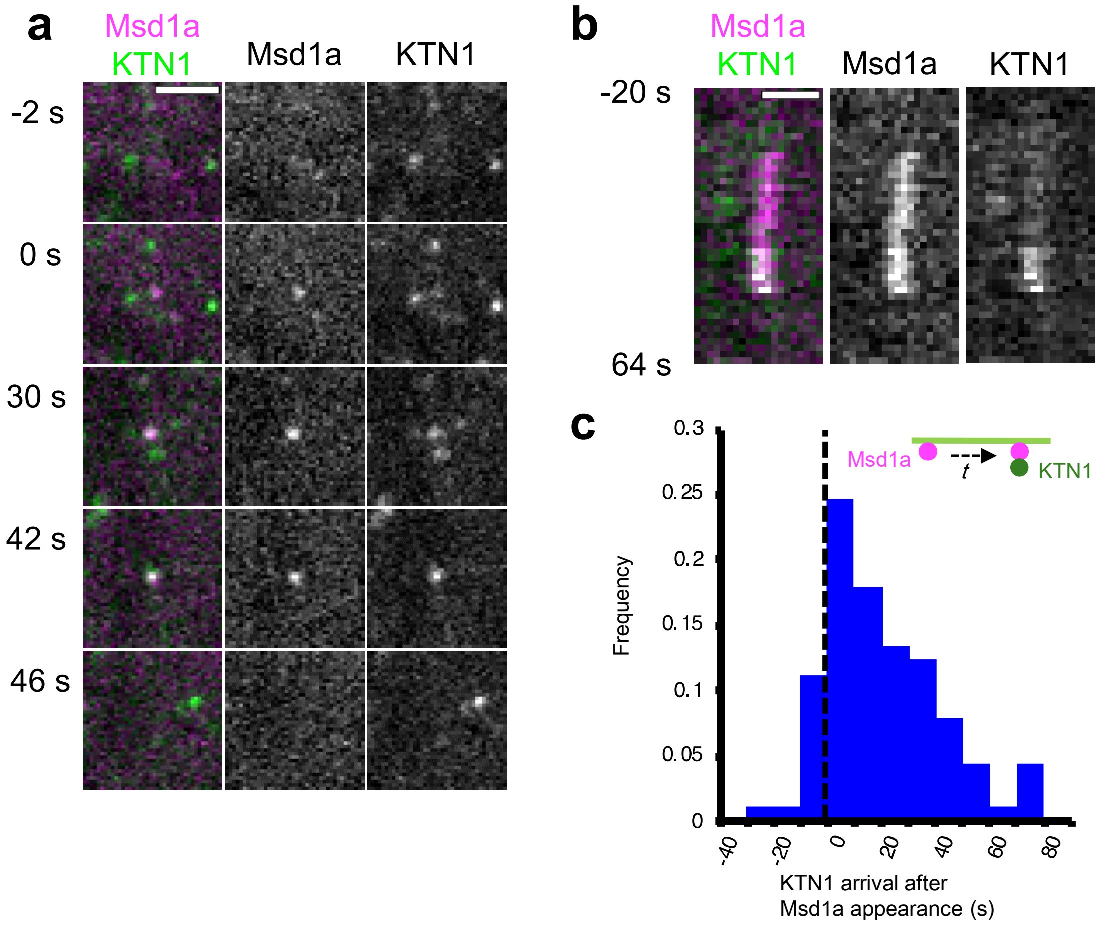

Supplementary Figure 4. The Msd1a particles arrive prior to the KTN1 particles. Recruitment of Msd1a-mCherry and GFP-KTN1 particles on the cell cortex region in Arabidopsis hypocotyl cells. **a**, Time-lapse confocal microscopy images at the indicated times. Bar, 2 µm. **b**, Kymograph generated from the time-lapse microscopy images shown in **a**. Bar, 1 µm. **c**, Distribution of the arrival times (*t* in the diagram) of Msd1a-mCherry particles (magenta) after the appearance of GFP-KTN1 particles (green) on the cell cortex (*n* = 90 events from 3 cells).

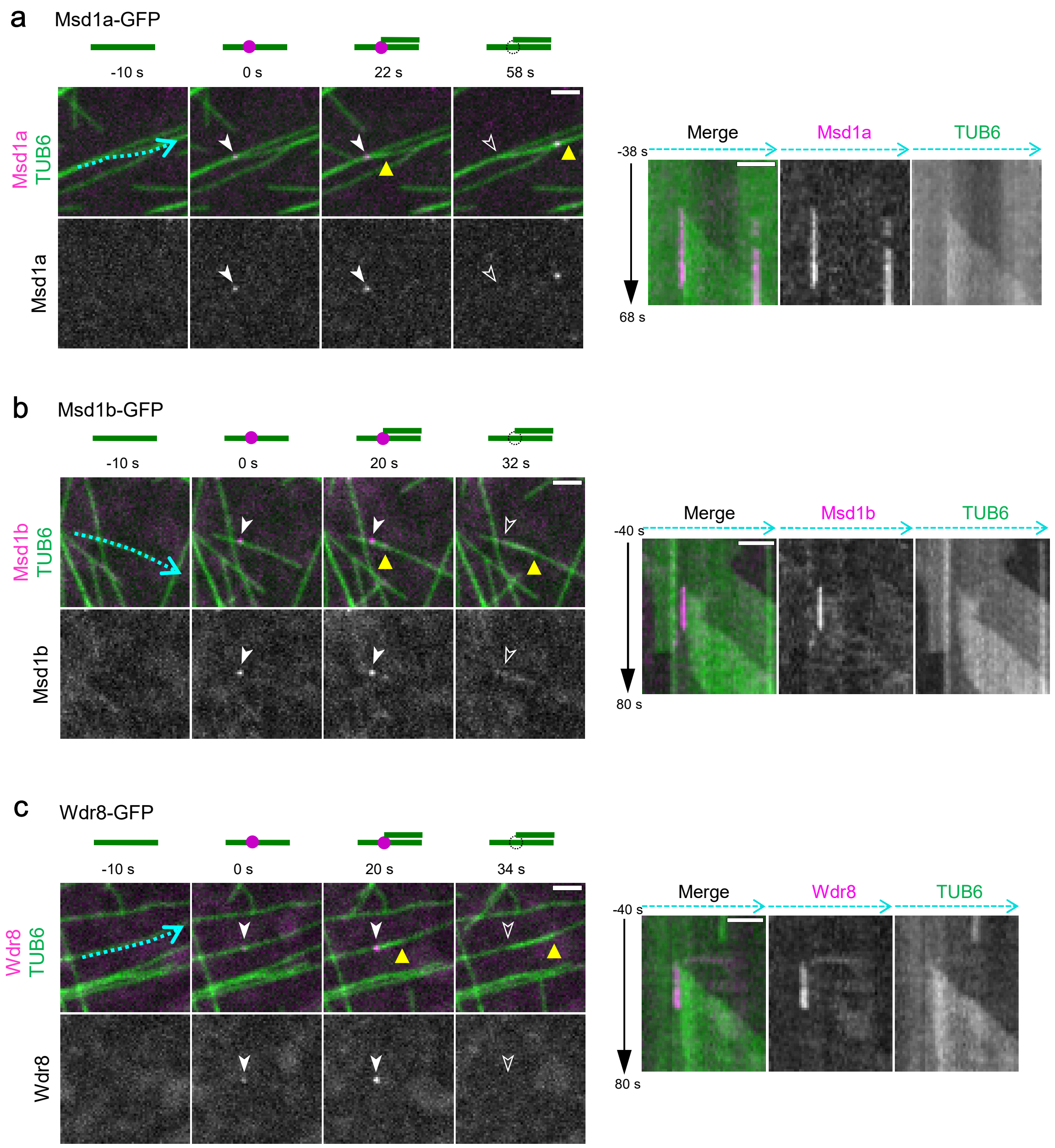

Supplementary Figure 5. Recruitment of Msd1a-GFP particles (a), Msd1b-GFP particles (b) and Wdr8-GFP particles (c) to the bundle-forming nucleation sites on cortical microtubules in wild-type cells. Time-lapse confocal microscopy images are shown at the indicated times. Kymographs were generated along the dotted blue. Open and closed arrowheads indicate the absence and the presence, respectively, of Msd1 or Wdr8 particles. Likewise, the yellow triangles show the plus ends of daughter microtubules. The events occurring at the indicated time points are schematically presented with microtubules (green lines) and Msd1-Wdr8 particles (magenta circles). Each panel is an averaged image of continuous 3 frames taken with 2 s-intervals. Bars, 2 µm.

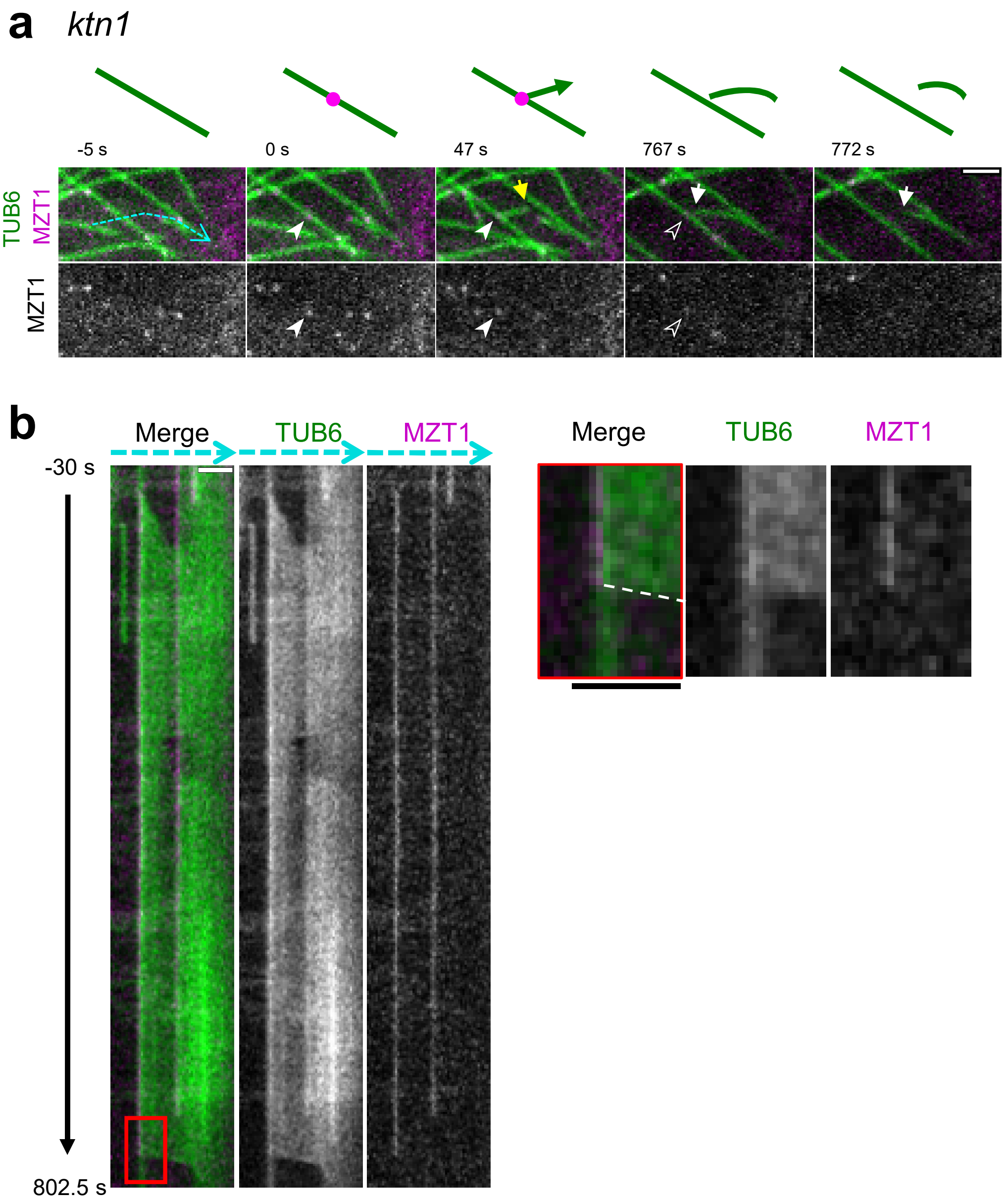

Supplementary Figure 6. Daughter microtubules are infrequently released long after nucleation from the branching nucleation sites in the *katanin* single mutant. **a**, Time-lapse confocal microscopy images of the minus-end release of daughter microtubules after branch-forming nucleation. γTuRC (magenta circles in the diagram) and microtubules (green lines) are labeled by MZT1-GFP and mCherry-TUB6, respectively. Open and closed arrowheads respectively indicate the absence and the presence of MZT1 particles, whereas yellow and white arrows show the plus and minus ends of daughter microtubules. **b**, A kymograph tracks the dotted blue line in **a**. Microtubule nucleation event sets the zero time point. The daughter microtubule is released at around 767s in the absence of katanin. The red boxed region is enlarged on the right. Bars, 2 μm.

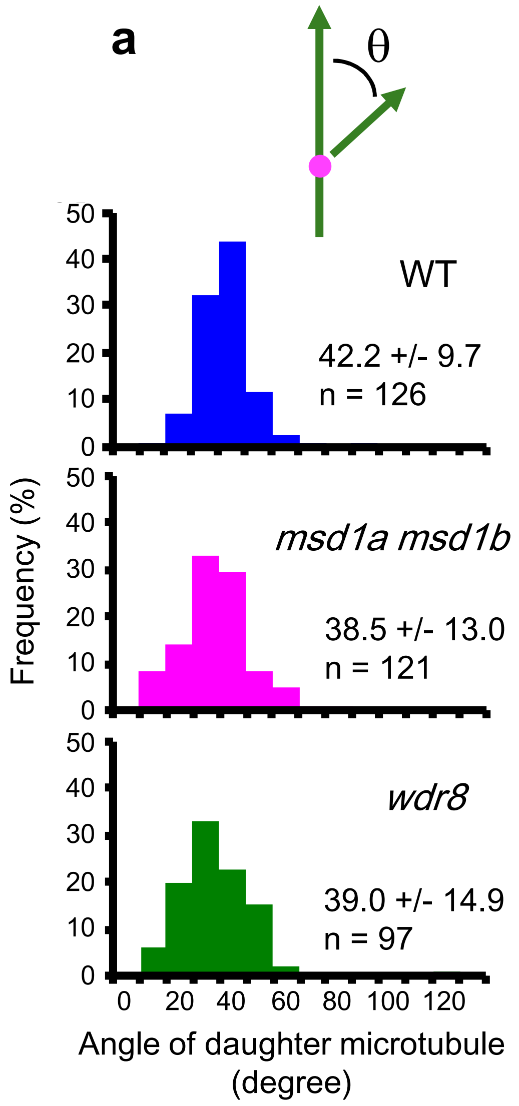

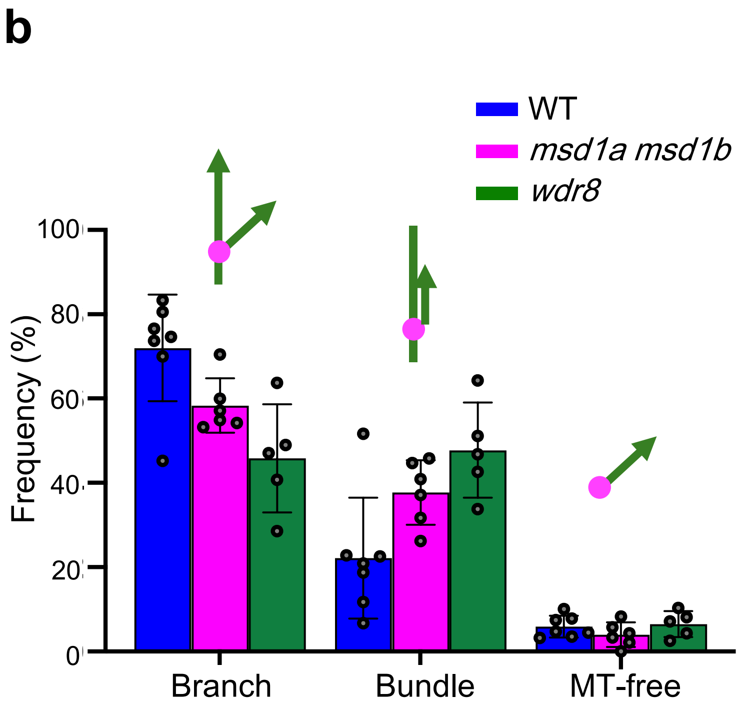

c

	Branch : Bundle	Branch : MT-free	Bundle : MT-free
WT : *msd1a msd1b*	7.80e-03	1	0.6377
WT : *wdr8*	5.77e-08	0.6377	0.668
msd1a msd1b : *wdr8*	1.07e-01	0.6377	1

Supplementary Figure 7. Microtubule nucleation characteristics. a, Angle (θ) of daughter microtubules relative to the mother microtubules in the branching nucleation. **b,** Classification of microtubule nucleation into the branching (Branch), bundle-forming (Bundle), and mother microtubule-independent (MT-free) types. The error bars indicate s.d. **c,** Statistical differences between frequencies of three nucleation types. Data are mean ± s.d. (*n* > 5 events). Dots represent exact data points of individual events. Pairwise multiple comparisons of pooled events were performed with Fisher's multcomp. function in R package, RVAideMemoire v 0.9-78 (https://www.rdocumentation.org/packages/RVAideMemoire/versions/0.9-78). For calculation of *p*-value, a Holm method with *p*.value.adjustment option was applied. Fisher's exact test shows that the branching nucleation is significantly decreased relative to the bundle-forming nucleation in *msd1* and *wdr8* cells compared to wild-type cells (*P*<0.001).

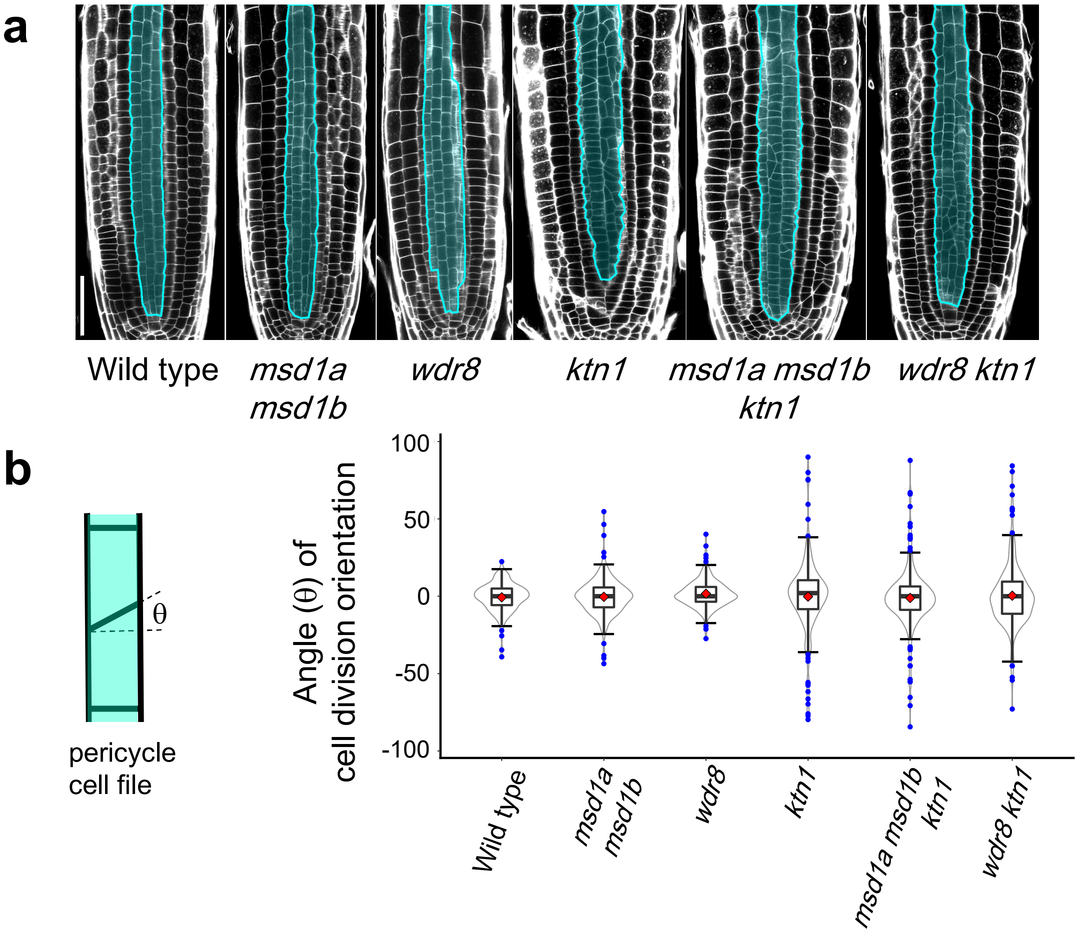

	Wild type	*msd1a msd1b*	*wdr8*	*ktn1*	*msd1a msd1b ktn1*	*wdr8 ktn1*
Mean	-0.7	-0.4	1.6	-0.2	-1.1	0.4
Variance (n-1)	85.1	150.8	84.6	595.3	417.4	427.2

c

	Wild type	*msd1a msd1b*	*wdr8*	*ktn1*	*msd1a msd1b ktn1*
Wild type	---	---	---	---	---
msd1a msd1b	0.849	---	---	---	---
wdr8	1.000	0.779	---	---	---
ktn1	<0.0001	<0.0001	<0.0001	---	---
msd1a msd1b ktn1	<0.001	<0.01	<0.0001	0.168	---
wdr8 ktn1	<0.0001	<0.001	<0.0001	0.686	0.926

Supplementary Figure 8. Mutations in *Msd1* and *Wdr8* do not complement the defects in cell division orientation of katanin mutant. a, Optical longitudinal sections of 4-day-old seedling roots of the indicated genotypes. Focal planes were set to highlight the pericycle cell layers (blue). Bar, 50 µm. **b**, Combined Tukey's box /violin plot diagram of division angles in cells of pericycle of wild type (153 cells from 3 plants), *msd1a msd1b* (192 cells from 3 plants), *wdr8* (157 cells from 3 plants), *ktn1* (195 cells from 3 plants), *msd1a msd1b ktn1* (207 cells from 3 plants), and *wdr8 ktn1* (229 cells from 3 plants). The mean and outliners are indicated as red diamonds and blue circles, respectively. The distance between the upper and lower whiskers shows the data range in each genotype. A schematic diagram on the left shows how the division angles (θ) are measured. Actual values of mean and variance are shown below the diagram. **c**, The Levene's statistics (*p* values) in its mean form for comparing variances between genotypes.

Supplementary Table 1. Primer sequences used in this study

Primer sequence (5'->3')	Purpose
gtgagaaacgtgaggctgatccttc<br>ctggaccatgctttcc	*msd1a-1* (SALK_148799) genotyping
atgccggcgaatgatgctg<br>ccaaatgtaaccatcgtct	*msd1a-2* (GABI_557F06) genotyping
atgtcattgactgtaccggag<br>gaatccagaggtgactaagc	*msd1b-1* (SALK_132426) genotyping
ccgaggttggaatatggacaac<br>gattcataatctccatgcctgatc	*msd1b-2* (GABI_596B10) genotyping
cgggtcctgaatcaccttacttgg<br>gtcatcagaactgtagtcgctgg	*wdr8-1* (SALK_093768) genotyping
gcgtggaccgcttgctgcaact	SALK line genotyping
cgacggatcgtaatttgtcg	GABI line genotyping
gtggacaaaaagaaagctgatggg<br>cttttagtcggaggattagactc	Msd1a genomic fragment cloning
ctcatgtaagaagccgcgac<br>ccacacatttttcgccagttag	Msd1b genomic fragment cloning
ctcaaagaaaaagtagatccgttc<br>ccacaagctacagagacttg	Wdr8 genomic fragment cloning
gtggacaaaaagaaagctgatggg<br>cttttagtcggaggattagactc	Msd1a cDNA cloning for yeast two-hybrid
ctcatgtaagaagccgcgac<br>ccacacatttttcgccagttag	Msd1b cDNA cloning for yeast two-hybrid
ctcaaagaaaaagtagatccgttc<br>ccacaagctacagagacttg	Wdr8 cDNA cloning for yeast two-hybrid
