# Supplementary material for: An anchoring complex recruits katanin for microtubule severing at the plant cortical nucleation sites
Source: Nat Commun. 2021 Jun 17;12:3687. doi: 10.1038/s41467-021-24067-y (PMC8211667; doi:10.1038/s41467-021-24067-y)
Supplement: Supplementary file 3 — Descriptions of Additional Supplementary Files [file 41467_2021_24067_MOESM3_ESM.pdf]

Descriptions of Additional Supplementary Files

Supplementary Movie 1
Description: Msd1a-GFP recruitment to a branching nucleation site. Time-lapse imaging of Msd1a-GFP (shown in magenta) and microtubules (shown in green) in a wild-type cotyledon pavement cell. Images were taken every 2 s. The appearance of Msd1a particle along mother microtubule lattice is set to 0 s. Open and close arrowheads respectively indicate the absence and the presence of Msd1a-GFP. The yellow and white triangles show the plus end and minus end of daughter microtubules, respectively. Scale bar, 2 µm. The Movie frame rate is 10 frames per s. Corresponding data is shown in Fig. 1c (left).

Supplementary Movie 2
Description: Absence of Msd1a-GFP puncta at a branching nucleation site in wdr8. Time-lapse imaging of Msd1a-GFP (shown in magenta) and microtubules (shown in green) in a wdr8 cotyledon pavement cell. Images were taken every 2 s. Microtubule nucleation is initiated at 0 s. Open and close arrowheads respectively indicate the absence and the presence of Msd1a-GFP. The yellow and white triangles show the plus end and minus end of daughter microtubules, respectively. Scale bar, 2 µm. The Movie frame rate is 10 frames per s. Corresponding data is shown in Fig. 1c (right)

Supplementary Movie 3
Description: Wdr8-GFP recruitment to a branching nucleation site. Time-lapse imaging of Wdr8- GFP (shown in magenta) and microtubules (shown in green) in a wild-type cotyledon pavement cell. Images were taken every 2 s. The appearance of Wdr8 particle along mother microtubule lattice is set to 0 s. Open and close arrowheads respectively indicate the absence and the presence of Wdr8-GFP. The yellow and white triangles show the plus end and minus end of daughter microtubules, respectively. Scale bar, 2 µm. The Movie frame rate is 10 frames per s. Corresponding data is shown in Fig. 1d (left).

Supplementary Movie 4
Description: Absence of Wdr8-GFP puncta at a branching nucleation site in msd1a msd1b. Timelapse imaging of Wdr8-GFP (shown in magenta) and microtubules (shown in green) in a msd1a msd1b cotyledon pavement cell. Images were taken every 2 s. Microtubule nucleation is initiated at 0 s. Open and close arrowheads respectively indicate the absence and the presence of Msd1a-GFP. The yellow and white triangles show the plus end and minus end of daughter microtubules, respectively. Scale bar, 2 µm. The Movie frame rate is 10 frames per s. Corresponding data is shown in Fig. 1d (right).

Supplementary Movie 5
Description: Msd1a-GFP particles on the cell cortex of wild-type cells. Localization of Msd1aGFP particles on the cell cortex region was followed over 302 s by time-lapse confocal microscopy. Kymograph analysis was performed along images of the cell cortex from the top to the bottom, as exemplified in Fig. 2a. Images were taken every 2 s. The Movie frame rate is 10 frames per s. Corresponding data is shown in Supplementary Figure 3a (WT).

Supplementary Movie 6
Description: Msd1a-GFP particles on the cell cortex of wdr8 cells. Localization of Msd1a-GFP particles on the cell cortex region was followed over 302 s by time-lapse confocal microscopy. Kymograph analysis was performed along images of the cell cortex from the top to the bottom, as exemplified in Fig. 2a. Images were taken every 2 s. The Movie frame rate is 10 frames per s. Corresponding data is shown in Supplementary Figure 3a (wdr8).

Supplementary Movie 7
Description: Wdr8-GFP particles on the cell cortex of wild-type cells. Localization of Wdr8-GFP particles on the cell cortex region was followed over 302 s by time-lapse confocal microscopy. Kymograph analysis was performed along images of the cell cortex from the top to the bottom, as exemplified in Fig. 2a. Images were taken every 2 s. The Movie frame rate is 10 frames per s. Corresponding data is shown in Supplementary Figure 3b (WT).

Supplementary Movie 8
Description: Wdr8-GFP particles on the cell cortex of msd1a msd1b cells. Localization of Wdr8- GFP particles on the cell cortex region was followed over 302 s by time-lapse confocal microscopy. Kymograph analysis was performed along images of the cell cortex from the top to the bottom, as exemplified in Fig. 2a. Images were taken every 2 s. The Movie frame rate is 10 frames per s. Corresponding data is shown in Supplementary Figure 3b (msd1a msd1b).

Supplementary Movie 9
Description: Msd1a-mCherry recruitment to the MZT1-GFP-labelled loci. Time-lapse imaging of Msd1a-mCherry (shown in magenta) and MZT1-GFP (shown in green) in a wild-type hypocotyl epidermal cell. Images were taken every 2.5 s. MZT1-GFP particle appearance to the cell cortex is observed at the time point zero. Blue triangle and white arrowhead respectively indicate MZT1-GFP and Msd1a-mCherry particles. Scale bar, 2 µm. The Movie frame rate is 5 frames per s. Corresponding data is shown in Fig. 1g.

Supplementary Movie 10
Description: Persistence of γTuRC labeled by MZT1-GFP on the cell cortex. Time-lapse imaging of MZT1-GFP in wild-type (upper) and msd1a msd1b (lower) cotyledon pavement cells. Images were taken every 2 s. Scale bar, 2 µm. The Movie frame rate is 20 frames per s. Corresponding data is shown in Fig. 2a.

Supplementary Movie 11
Description: Daughter microtubule nucleation and release at the cell cortex in a wild-type cell. Time-lapse imaging of MZT1-GFP (shown in magenta) and microtubules (shown in green) in a wild-type cotyledon pavement cell. Images were taken every 2 s. MZT1-GFP particle appearance on mother microtubule lattice is set to 0 s. Open and close arrowheads respectively indicate the absence and the presence of MZT1-GFP. The yellow and white triangles show the plus end and minus end of daughter microtubules, respectively. Scale bar, 2 µm. The Movie frame rate is 10 frames per s. Corresponding data is shown in Fig. 2d.

Supplementary Movie 12
Description: Complete shrinkage event of daughter microtubule at the cell cortex in a wild-type cell. Time-lapse imaging of MZT1-GFP (shown in magenta) and microtubules (shown in green) in a wild-type cotyledon pavement cell. Images were taken every 2 s. MZT1-GFP particle appearance on mother microtubule lattice is set to 0 s. Arrowhead and yellow triangle indicate the presence of MZT1-GFP and the plus end of daughter microtubules, respectively. Scale bar, 2 µm. The Movie frame rate is 10 frames per s. Corresponding data is shown in Fig. 2e.

Supplementary Movie 13
Description: GFP-KTN1 recruitment to Msd1a-mCherry-labeled loci. Time-lapse imaging of Msd1a-mCherry (shown in magenta) and GFP-KTN1 (shown in green) in a wild-type hypocotyl epidermal cell. Images were taken every 2 s. Msd1a-mCherry particle appearance to the cell cortex is set to 0 s. Arrowhead and blue triangle indicate Msd1a- mCherry and GFP-KTN1 particles, respectively. Scale

bar, 2 µm. The Movie frame rate is 5 frames per s. Corresponding data is shown in Supplementary Figure 4a.

Supplementary Movie 14
Description: Katanin recruitment to and subsequent release of branched daughter microtubule in a wild-type cell. Time-lapse imaging of GFP-KTN1 (shown in magenta) and microtubules (shown in green) in a wild-type cotyledon pavement cell. Images were taken every 2 s. Daughter microtubule nucleation is initiated at 0 s. Open and close arrowheads indicate the absence and the presence of GFP-KTN1 at the nucleation site, respectively. The yellow and white arrows show the plus end and minus end of daughter microtubules, respectively. Scale bar, 2 µm. The Movie frame rate is 10 frames per s. Corresponding data is shown in Fig. 3a.

Supplementary Movie 15
Description: Release of branched daughter microtubule without KTN1 recruitment to the nucleation site in a wdr8 cell. Time-lapse imaging of GFP-KTN1 (shown in magenta) and microtubules (shown in green) in a wdr8 cotyledon pavement cell. Images were taken every 2 s. Daughter microtubule nucleation is initiated at 0 s. Open arrowheads indicate the absence of GFP-KTN1 at the nucleation site. The yellow and white arrows show the plus end and the minus end of daughter microtubules, respectively. Closed blue arrowhead points to GFP-KTN1 at the microtubule crossover site. Scale bar, 2 µm. The Movie frame rate is 10 frames per s. Corresponding data is shown in Fig. 3c.

Supplementary Movie 16
Description: Msd1a-GFP recruitment to a bundle-forming nucleation site. Time-lapse imaging of Msd1a-GFP (shown in magenta) and microtubules (shown in green) in a wild-type cotyledon pavement cell. Images were taken every 2 s. The appearance of Msd1a particle along mother microtubule lattice is set to 0 s. Open and close arrowheads respectively indicate the absence and the presence of Msd1a-GFP. The yellow and white triangles show the plus end and minus end of daughter microtubules, respectively. Scale bar, 2 µm. The Movie frame rate is 10 frames per s. Corresponding data is shown in Supplementary Figure 5a.

Supplementary Movie 17
Description: Msd1b-GFP recruitment to a bundle-forming nucleation site. Time-lapse imaging of Msd1b-GFP (shown in magenta) and microtubules (shown in green) in a wild-type cotyledon pavement cell. Images were taken every 2 s. The appearance of Msd1b particle along mother microtubule lattice is set to 0 s. Open and close arrowheads respectively indicate the absence and the presence of Msd1b-GFP. The yellow and white triangles show the plus end and minus end of daughter microtubules, respectively. Scale bar, 2 µm. The Movie frame rate is 10 frames per s. Corresponding data is shown in Supplementary Figure 5b.

Supplementary Movie 18
Description: Wdr8-GFP recruitment to a bundle-forming nucleation site. Time-lapse imaging of Wdr8-GFP (shown in magenta) and microtubules (shown in green) in a wild-type cotyledon pavement cell. Images were taken every 2 s. The appearance of Wdr8 particle along mother microtubule lattice is set to 0 s. Open and close arrowheads respectively indicate the absence and the presence of Wdr8-GFP. The yellow and white triangles show the plus end and minus end of daughter microtubules, respectively. Scale bar, 2 µm. The Movie frame rate is 10 frames per s. Corresponding data is shown in Supplementary Figure 5c.

Supplementary Movie 19
Description: Katanin recruitment to and subsequent release of bundled daughter microtubule in a

wild-type cell. Time-lapse imaging of GFP-KTN1 (shown in magenta) and microtubules (shown in green) in a wild-type cotyledon pavement cell. Images were taken every 2 s. Daughter microtubule nucleation is initiated at 0 s. Open and close arrowheads indicate the absence and the presence of GFP-KTN1 at the nucleation site, respectively. The yellow and white arrows show the plus end and minus end of daughter microtubules, respectively. Scale bar, 2 µm. The Movie frame rate is 10 frames per s. Corresponding data is shown in Fig. 3f.

Supplementary Movie 20
Description: Release of bundled daughter microtubule without KTN1 recruitment to the nucleation site in a wdr8 cell. Time-lapse imaging of GFP-KTN1 (shown in magenta) and microtubules (shown in green) in a wdr8 cotyledon pavement cell. Images were taken every 2 s. Daughter microtubule nucleation is initiated at 0 s. Open arrowheads indicate the absence of GFP-KTN1 at the nucleation site. The yellow and white arrows show the plus end and the minus end of daughter microtubules, respectively. Scale bar, 2 µm. The Movie frame rate is 10 frames per s. Corresponding data is shown in Fig. 3g.

Supplementary Movie 21
Description: Microtubule nucleation event in a ktn1 cell. Time-lapse imaging of MZT1-GFP (shown in magenta) and microtubules (shown in green) in a ktn1 cotyledon pavement cell. Images were taken every 2.5 s. MZT1-GFP particle appearance on mother microtubule lattice is set to 0 s. Arrowheads indicate MZT1-GFP at the nucleation site. The yellow arrow shows the plus end of daughter microtubules. Scale bar, 2 µm. The Movie frame rate is 20 frames per s. Corresponding data is shown in Fig. 4a.

Supplementary Movie
Description: 22 Daughter microtubule nucleation and release at the cell cortex in a ktn1 cell. Timelapse imaging of MZT1-GFP (shown in magenta) and microtubules (shown in green) in a ktn1 cotyledon pavement cell. Images were taken every 2.5 s. MZT1-GFP particle appearance on mother microtubule lattice is set to 0 s. Open and close arrowheads indicate the absence and the presence of MZT1-GFP, respectively. The yellow and white arrows show the plus end and the minus end of daughter microtubules, respectively. Scale bar, 2 µm. The Movie frame rate is 10 frames per s. Corresponding data is shown in Supplementary Figure 6.

Supplementary Movie 23
Description: Daughter microtubule nucleation and release at the cell cortex in a wdr8 ktn1 cell. Time-lapse imaging of MZT1-GFP (shown in magenta) and microtubules (shown in green) in a wdr8 ktn1 cotyledon pavement cell. Images were taken every 2.5 s. MZT1- GFP particle appearance on mother microtubule lattice is set to 0 s. Open and close arrowheads indicate the absence and the presence of MZT1-GFP, respectively. The yellow and white arrows show the plus end and the minus end of daughter microtubules, respectively. Scale bar, 2 µm. The Movie frame rate is 10 frames per s. Corresponding data is shown in Fig. 4b.

Supplementary Movie 24
Description: Subcellular localization of katanin and Msd1 in mitotic cells. Time-lapse imaging of GFP-KTN1 (shown in green) and Msd1a-mCherry (shown in magenta) in wild-type root epidermal cells. Images were taken every 30 s. Nuclear envelope breakdown of upper left cell (yellow square) is set to 00:00 (minute:second). Scale bar, 10 µm. The Movie frame rate is 10 frames per s. Corresponding data is shown in Fig. 5a.

Supplementary Movie 25
Description: Subcellular localization of katanin in a dividing wild-type cell. Time-lapse imaging of GFP-KTN1 in wild-type root epidermal cells. Images were taken every 30 s. Each frame is an averaged image of 3 z-slices taken with 0.7 µm step. Beginning of the nuclear envelop breakdown is set to 00:00 (minute:second). Scale bar, 5 µm. The Movie frame rate is 10 frames per s. Corresponding data is shown in Fig. 5b.

Supplementary Movie 26
Description: Subcellular localization of katanin in a dividing wdr8 cell. Time-lapse imaging of GFP-KTN1 in wdr8 root epidermal cells. Images were taken every 30 s. Each frame is an averaged image of 3 z-slices taken with 0.7 µm step. Beginning of the nuclear envelop breakdown is set to 00:00 (minute:second). Scale bar, 5 µm. The Movie frame rate is 10 frames per s. Corresponding data is shown in Fig. 5b.

Supplementary Movie 27
Description: Katanin recruitment to a microtubule crossover site and subsequent severing at the cell cortex of a wild-type cell. Time-lapse imaging of GFP-KTN1 (shown in magenta) and microtubules (shown in green) in a wild-type cotyledon pavement cell. Images were taken every 2 s. Microtubule crossover formation where microtubule severing was subsequently observed set the time point zero. Open and close arrowheads respectively indicate the absence and the presence of GFP-KTN1 at the crossover site. The yellow and blue arrows respectively show the plus end and the minus end of the focused microtubules, while the white arrow indicates the shrinking plus end of a severed microtubule. Scale bar, 2 µm. The Movie frame rate is 10 frames per s. Corresponding data is shown in Fig. 6a.

Supplementary Movie 28
Description: Katanin recruitment to a microtubule crossover site and subsequent severing at the cell cortex of a wdr8 cell. Time-lapse imaging of GFP-KTN1 (shown in magenta) and microtubules (shown in green) in a wdr8 cotyledon pavement cell. Images were taken every 2 s. Microtubule crossover formation where microtubule severing was subsequently observed set the time point zero. Open and close arrowheads respectively indicate the absence and the presence of GFP-KTN1 at the crossover site. The yellow and blue arrows respectively show the plus end and the minus end of the focused microtubule, while the white arrow indicate the shrinking plus end of a severed microtubule. Scale bar, 2 µm. The Movie frame rate is 10 frames per s. Corresponding data is shown in Fig. 6b.

Supplementary Movie 29
Description: Microtubule severing at the crossover site in the absence of Msd1. Time-lapse imaging of Msd1a-GFP (shown in magenta) and microtubules (shown in green) in a wildtype cotyledon pavement cell. Images were taken every 2 s. Microtubule nucleation initiates at the time point zero. Open and close arrowheads respectively indicate the absence and the presence of Msd1a-GFP at the crossover site and the nucleation site. Scale bar, 2 µm. The Movie frame rate is 10 frames per s. Corresponding data is shown in Fig. 6e.

Supplementary Movie 30
Description: Microtubule severing at the crossover site in the absence of Wdr8. Time-lapse imaging of Wdr8-GFP (shown in magenta) and microtubules (shown in green) in a wildtype cotyledon pavement cell. Images were taken every 2 s. Microtubule nucleation initiates at the time point zero. Open and close arrowheads respectively indicate the absence and the presence of Wdr8-GFP at the

crossover site and the nucleation site. Scale bar, 2 µm. The Movie frame rate is 10 frames per s. Corresponding data is shown in Fig. 6f.
